# Moral contingentism, personites and countermoral worlds

**DOI:** 10.1007/s11229-026-05725-4

**Published:** 2026-07-21

**Authors:** Tommaso Soriani

**Affiliations:** Department of Philosophy, University of Reading, Shinfield Road, Reading, RG6 6EL UK

**Keywords:** Perdurantism, Personites, Duplication, Moral contingentism, Normative necessity

## Abstract

According to Mark Johnston’s Personite Problem, Perdurantism—the view that persons are maximal fusions of person-stages—should be rejected, as it fails to account for the moral status of non-maximal fusions that overlap a person (*personites*). His Duplication Argument claims that personites have persons as duplicates in other metaphysically possible worlds. Since duplicates share all intrinsic descriptive properties, they must share normative ones, including moral status. I challenge this by accepting Johnston’s duplication claim but rejecting its moral conclusion. Perdurantists can respond by adopting Moral Contingentism: the view that pure moral laws are not *metaphysically necessary* but only *normatively necessary*, hence metaphysically contingent. These perdurantists can maintain metaphysically possible worlds where personites have person-duplicates, while holding these worlds normatively impossible (i.e., *countermoral*). Countermoral worlds—differing in their pure moral laws and thus how they ground moral status—cannot confer moral status on personites through duplication in normatively possible worlds, including the actual one.

## Introduction

Mark Johnston’s Personite Problem holds that Perdurantism, according to which persons are maximal fusions of person-stages, should be rejected as it fails to account for the moral status of non-maximal fusions that overlap significant portions of a person’s lifetime, personites. Its main argument, the Duplication Argument, claims that personites have persons as duplicates in other possible worlds; and since duplicates share all intrinsic descriptive properties, they must also share normative ones, including moral status.

In this paper, I argue that the Duplication Argument can nonetheless be resisted by perdurantists who adopt Moral Contingentism, even while accepting Johnston’s claim that personites have possible persons as duplicates. The view holds that pure moral laws are not metaphysically necessary but contingent, while still being normatively necessary, a weaker form of necessity. Such perdurantists can maintain that there is a metaphysically possible world in which a duplicate of a personite is a person while also holding that this world is normatively impossible, and therefore very distant in normative space, i.e., a countermoral world. Such a world cannot confer moral status on a personite in normatively possible worlds, including the actual world, since the pure moral laws there may differ and ground it differently.

In Sect. 2, I provide an overview of Johnston’s Personite Problem and then focus on the Duplication Argument. Then, after introducing the distinction between normative and descriptive properties, and discussing the possible metaphysical dependencies of the former on the latter (Sect. 3), I turn in Sect. 4 to Moral Contingentism, focusing on its central tenets. Section 5 presents my Moral Contingentist Counterargument to Johnston’s Duplication Argument. In Sect. 6, I respond to several concerns related to Moral Contingentism and my counterargument. I address the Authority Objection, which targets views like Moral Contingentism by arguing that, since pure moral laws are not metaphysically necessary, they cannot fulfill an action-guiding role. However, I focus primarily on the Luck Objection, which holds that Moral Contingentism renders every moral deliberation and action vulnerable to moral luck, as one’s location in a particular world, given the metaphysical contingency of pure moral laws, seems to be a matter of luck.

## Perdurantism and the personite problem: Johnston’s duplication argument

Perdurantists hold that persons are four-dimensional entities, more precisely maximal mereological fusions of R-interrelated^1^ instantaneous temporal parts—person-stages—extended over a time interval—their lifetime.^2^ Additionally, perdurantists often endorse Unrestricted Composition, the mereological principle which states that for any arbitrary collection of objects, there exists a fusion.^3^

Applied to persons, this implies that we can “slice them up” and obtain as many non-maximal fusions of R-interrelated temporal parts as we like, each overlapping with the person over some interval within her lifetime. Many of these non-maximal fusions qualify as what Johnston calls personites: they overlap with a person for a significant interval of time but, unlike the person with whom they overlap, they are denied moral status. To those who argue that their prudential interests need not be considered, since they align with the person’s, Johnston responds that personites are in a state of *false consciousness*: they believe they are the person because they share the *same* numerically identical mind and, consequently, the *same* thoughts,^4^ yet their true interests diverge (Johnston, 2017, p. 632).

Consider, for example, a seemingly harmless action, or at least one that does not potentially harm anyone except perhaps the individual willingly choosing to do it, such as enrolling in an intensive Hungarian language course to prepare for an upcoming trip to Budapest. According to Johnston, under Perdurantism, this action is far from innocent. Many personites endure the hardship of learning Hungarian only to cease to exist before the person reaches Budapest. Johnston’s argument is that, for these short-lived personites, taking the course is not in their best interest. Consequently, Perdurantism appears morally untenable, as it entails the suffering of many entities who are never compensated for their efforts, effectively disregarding their moral status. Mutatis mutandis, similar considerations apply to nearly every other normative practice, such as punishing, promising, and so on: this is Johnston’s Personite Problem.^5^

For Johnston, personites are denied moral status mainly due to an extrinsic factor: they are not maximal but merely a large proper part of a maximal fusion of R-interrelated stages. If there were no stages after them, they would attain maximality and, consequently, personhood and moral status. Johnston’s main argument, the Duplication Argument, hinges precisely on this last counterfactual point: personites have possible persons as duplicates, and since duplicates share the same intrinsic *descriptive* properties, they must also share moral status, given that this *normative* property depends on the former:

Imagine the case of Dum and Dee, two identical twins […] Dum dies […] half an hour before Dee dies […] The sum of stages that is Dee, minus those stages that make up the last half hour of Dee’s life, is a personite. Still, that personite—“Dee-minus”—*could be* intrinsically just like the person Dum. Dee-minus fails to deserve the name of a person only because of what happened after it ceased to exist. Dee-minus is not a person. But Dee-minus, being intrinsically like the person Dum, does seem to be sufficiently person-like to deserve the respect that we extend to persons. (Johnston, 2017, p. 619, emphasis mine).

Some clarifications are in order. Prima facie, the way Johnston presents it, Dum and Dee-minus appear to be duplicates at the very same world: their lifetimes are equally extended, and, he claims, they seem to share all their intrinsic descriptive properties. However, if Dum has any duplicates at her world, Dee, her twin who dies 30 min later, seems to be a more suitable candidate. After all, the fact that Dee dies 30 min later than Dum appears to be something extrinsic to her description, as Johnston himself recognizes, whereas the property of dying, which Dee-minus does not have, since it^6^ simply ceases to exist—viz., it does not include a dying stage, unlike Dum and Dee—seems to be a candidate intrinsic descriptive property. This, along with other candidate intrinsic properties more or less directly related to death and to what occurs before and after it, may give us reason to reject Dee-minus as a suitable duplicate of Dum. Furthermore, the idea that personites can have persons inhabiting their same world as duplicates—and, more generally, that world-mates need only share their intrinsic properties to qualify as duplicates—proves problematic upon reflection. Accordingly, and this will become more relevant shortly, I will assume that if personites can have persons as duplicates, these persons must be located in other worlds.^7^

The point, in fact, is that there is a sense in which the counterfactual under examination, something like “If Dee died 30 minutes earlier, then Dee-minus would have been a person”, could be true. It seems possible that Dee could have died 30 min later, as Dum did, and so, if not Dee-minus for the reasons just mentioned, then something very much like it would have been the maximal fusion of stages and therefore the person. The overall closeness of such a possible world is debatable; but if the counterfactual is true, then it must be the closest world in which Dee dies 30 min earlier. And it does seem that we can conceive of such a world, however remote it may be.^8^  

Taking all this into account, I align with Johnston in accepting that a counterpart of Dee-minus, whether maximal or not, at another world could be a duplicate of Dum, and, more generally, that personites can have persons as duplicates, provided they are not world-mates. With this clarified, Johnston’s Duplication Argument can be generalized as follows:^9^

1. (All persons have moral status as a matter of metaphysical necessity): For all metaphysically possible worlds *w* and possible objects *x*, if *x* is a person in *w* then *x* has moral status in *w*.
2. (All duplicates share moral status): For all metaphysically possible worlds w and v and possible objects *x* and *y*, if *x* in *w* is a duplicate of *y* in *v*, then *x* has moral status in *w* iff *y* has moral status in *v*.
3. (All personites have at least one possible person as a duplicate as a matter of metaphysical necessity): For all personites *x*, there is a metaphysically possible object *y* and a possible world *w*, distinct from the actual one, such that *y* is a person in *w* and *y* in *w* is a duplicate of *x* in the actual world.

Therefore: All personites have moral status as a matter of metaphysical necessity.

Notable challenges to the Duplication Argument have been advanced by Kowalczyk (2022) and Dorr and Hawthorne (forthcoming). Dorr and Hawthorne question whether personites really qualify as duplicates of persons, especially once one examines the role of intrinsic and modal properties in the duplication relation. Kowalczyk, by contrast, proposes restricting the duplication principle so that large proper parts such as personites need not inherit all the intrinsic properties of the wholes in which they are embedded.^10^ In this paper, I explore whether the Duplication Argument can be blocked by adopting a different and novel metaethical strategy: the adoption of Moral Contingentism by perdurantists. According to this view, pure moral laws are metaphysically contingent while remaining normatively necessary. Thus, it is metaphysically possible for two things to be alike in every non-moral respect and yet differ morally, relative to a given set of pure moral laws (Rosen, 2020; Väyrynen, 2021).

My Moral Contingentist Counterargument to Johnston’s Duplication Argument avoids any commitment about which intrinsic descriptive properties differ between persons and personites, unlike the strategies mentioned above. Perdurantists who adopt Moral Contingentism can hold that, despite them being descriptively identical, persons have moral status and personites do not as a matter of normative necessity, due to the pure moral laws in place which ground moral status in this way. This allows them to retain the metaphysical possibility of worlds in which personites such as Dee-minus have persons as duplicates, while claiming that such worlds lie outside the set of normatively possible worlds: they are *countermoral worlds*, since the pure moral laws can differ there and ground moral status differently. As a result, they can conclude that personites also fail to acquire moral status through duplication, resisting Johnston’s conclusion that they have it as a matter of metaphysical necessity.

## Normative and descriptive properties, and metaphysical dependencies

First of all, a gloss on normative and descriptive properties and their relation is needed. On the one hand, descriptive properties are generally understood as physical or psychological characteristics, and sometimes as *natural*—although this latter commitment may not be strictly required—features that can be described without reference to normative concepts such as good, right, or just. On the other hand, normative properties, like the ones just mentioned, usually reference descriptive properties in their explanation (cf. Streumer, 2011, pp. 326–328). While descriptive properties may not fully explain normative properties, they must at least partially figure in their explanation, at least those that are intrinsic; for simplicity, I will use “descriptive properties” to refer only to intrinsic ones from now on.

Consider the following: under Act-Consequentialism, an act is defined as good if it maximizes well-being, which, if we are pluralists, can take various forms. In any case, it is ultimately explained by descriptive properties obtaining, such as pleasure, desire-satisfaction, or the absence of suffering. This pattern is not unique to Act-Consequentialism; all other ethical theories rely on descriptive properties to support their normative claims. For instance, in Kantian Deontology, an act is good if it adheres to certain duties or rules, such as telling the truth or respecting autonomy. Similarly, Virtue Ethics ties moral goodness to the cultivation of virtues like courage, honesty, or compassion, which are understood in terms of non-normative traits, such as emotional dispositions, behavioral patterns, or psychological states that contribute to human flourishing.

Similar considerations apply specifically to *moral status*: the fact that an entity possesses moral status is usually *explained* by certain descriptive properties that the entity has. These may include cognitive capacities related to consciousness, such as self-awareness, rationality, and so on. Even in cases where such capacities are diminished or absent, such as in comatose individuals, the potential for cognition or past possession of these capacities can play a role. Alternatively, moral status might simply rest on more basic features, such as the capacity to feel pain.^11^ In any case, the attribution of moral status, and of normative properties in general, appears to *depend* not just conceptually but also *metaphysically* on descriptive properties that, when instantiated, give rise to normative considerations. The strongest way to capture this dependence, understood throughout this paper in terms of *grounding*, is through a principle along the following lines:

**Full Metaphysical Dependence of the Normative (FMD)**: As a matter of metaphysical necessity, every normative property is *fully* grounded in one or more descriptive properties (cf. Bader, 2017, p. 117; Morton, 2020, p. 174).

This is a principle that most metaethical positions would endorse, including those Moral Realists who identify as *naturalists*, and it is central to understanding the relationship between normative and descriptive properties. The clause of metaphysical necessity is included to capture a widely held assumption: that the dependence of normative properties on descriptive properties holds across all metaphysically possible worlds, because moral laws are taken to be metaphysically necessary. This is often regarded as a desirable feature of morality for two main reasons. First, if moral laws were not metaphysically necessary, they would, according to proponents of this view, lack the authoritative force required to guide our actions. Second, if moral laws were not metaphysically necessary, then there could be two metaphysically possible worlds that are descriptive duplicates yet governed by different moral laws. This would not only undermine the action-guiding function of morality in our world but could also threaten the justification of our moral beliefs, and thereby our moral knowledge—naturally, the significance of this last concern will vary across metaethical views, depending on how much emphasis they place on the justification of moral beliefs and on moral knowledge more generally.^12^

So, going back to the Duplication Argument, a perdurantist who endorses FMD has no choice but to try to block the argument by arguing that personites do not have possible persons as duplicates. Another option is to reject FMD but then there still is its weaker version:

**Partial Metaphysical Dependence of the Normative (PMD)**: As a matter of metaphysical necessity, every normative property is *partially* grounded in one or more descriptive properties (cf. Bader, 2017, p. 117; Morton, 2020, p. 176).

PMD alone does not offer the perdurantist an escape. If the remaining grounds of moral status are themselves metaphysically necessary—for instance, as moral laws are under Moral Necessitism—then duplicates continue to share moral status, and the Duplication Argument retains its full force. Rejecting PMD is not a viable option either, since it would sever any grounding connection between moral status and descriptive properties. This would be a highly revisionary commitment: whatever grounds moral status would then be independent, in the broadest possible sense, from descriptive properties. The proponent of such a view thus treats the obtaining of moral status as a *brute* fact, leaving unexplained why persons and personites differ in moral status.^13^

The upshot is that PMD should be retained, but it is not sufficient on its own. What is needed, in addition to PMD, is the further claim that the remaining grounds of moral status are metaphysically contingent. This is precisely the main thesis of Moral Contingentism, which takes those remaining grounds to be metaphysically contingent pure moral laws, as will be introduced in the following section.

## Moral contingentism, pure moral laws and grounding

The only additional thing that could explain the difference in moral status between Dum and Dee-minus would be an additional element in which Dum’s moral status is grounded. However, this element must not perform the same grounding work for Dee-minus — which is why Dee-minus fails to have moral status. So, it cannot be descriptive by stipulation, and it cannot be grounded in itself, as this would violate irreflexivity.

A candidate for this element is what Rosen (2017, 2020, 2021) along with others (Väyrynen, 2021) refer to as a *pure* moral law, so something that is both normative and *necessary*—I will explain in a moment in what sense. Examples of pure moral laws include the Greatest Happiness Principle of Consequentialism, Kant’s Categorical Imperative, or the Golden Rule, to mention some of the less controversial instances.^14^ The main feature of pure moral laws is that they hold independently of any descriptive fact, as I will also clarify shortly.

But first, let me remark that, to avoid blocking the argument too early, it is important to preserve the counterfactual duplication-based point mentioned at the beginning. Thus, the pure moral law in question must be sufficiently modally robust to confer moral status to persons not only in the actual world but also in nearby possible worlds, while at the same time denying it to personites. Yet it cannot be too modally robust, as it would be under Moral Necessitism, since it must also allow for the metaphysical possibility of a world in which Dee-minus has a possible person as duplicate without giving it moral status in the actual world.

This is where Moral Contingentism comes to the rescue: pure moral laws are not metaphysically necessary but only *normatively necessary*, making it metaphysically possible for two things to be alike in every descriptive respect and yet differ normatively. Following Fine (2020) and Rosen (2021), normative necessity is taken to be “weaker” than metaphysical necessity in that it takes less to break: like nomological necessities, normative ones can fail in metaphysically possible worlds, whereas metaphysical necessities should not.^15^

A useful way to further understand the relationship between normative and metaphysical necessity is, in fact, to compare it to the link between nomological and metaphysical necessity. Just as a world can be metaphysically possible but nomologically impossible, a world can also be metaphysically possible but normatively impossible. For example, imagine a world where a macroscopic object moves faster than light: this would violate our laws of nature, making it nomologically impossible, but there is no deeper metaphysical contradiction in the concept of superluminal travel, so the world remains metaphysically possible. Similarly, we could imagine a metaphysically possible world where torturing others for fun is permissible. If permissibility is tied to specific pure moral laws in place in the actual world, then such a world would be normatively impossible, because it contradicts our principles of permissibility.^16^

So, the key feature of Moral Contingentism is what Rosen (2020, p. 219) calls the *fact-independence* of normatively necessary propositions such as pure moral laws:^17^ within any given non-empty subset of the metaphysically possible worlds—the normatively possible ones—these laws—e.g., those constituting Act-Consequentialism—hold independently of any descriptive fact. Moreover, since pure moral laws are metaphysically contingent, it is metaphysically possible that in our world, @, Act-Consequentialism is true, while in world *w* Kantian Deontology is true, and yet the two worlds are alike in every descriptive respect, differing normatively in virtue of different pure moral laws: *w* and @ are normatively impossible, and thus countermoral worlds, with respect to each other, and so distant in normative space.^18^ For example, in @ it is sometimes right to lie to spare someone from harm, whereas in *w* it is always wrong.

According to what has been said so far, Moral Contingentism involves the adoption of something like the following dependence principle:

**Normative Dependence on Pure Moral Laws (ND)**: As a matter of normative necessity, some^19^ normative properties are *partially* grounded in one or more pure moral laws (cf. Morton, 2020, p. 191).

If *A* (e.g., one or more pure moral laws) and *C* (one or more descriptive properties, e.g., consciousness and/or the capacity to feel pain)^20^ together fully ground *B* (e.g., moral status) as a matter of normative necessity, then *B* obtains in all possible worlds where *A* and *C* are present, the normative possible worlds, but it does not follow that it obtains in all metaphysically possible worlds. The metaphysically possible worlds outside the normatively possible ones—in which *B* does not obtain or is grounded differently—are classified as normatively impossible, i.e., countermoral worlds. Moreover, as already remarked, some worlds that are countermoral worlds relative to each other may be descriptively identical and yet differ normatively due to different pure moral laws holding there.

To recap, in Moral Contingentism, moral status and similar properties are normative *because* they are partially grounded in one or more pure moral laws as a matter of normative necessity. What has been described as a feature of the view, that whether any particular pure moral law obtains is a metaphysically contingent matter, has a cost: it makes moral status metaphysically contingent, thereby also allowing two descriptive duplicates located at different metaphysical possible worlds to differ with respect to it. For perdurantists who wish to challenge the Duplication Argument’s moral conclusion without abandoning the metaphysical possibility that personites could have persons as duplicates, however, this cost may well be worth paying, as the next section aims to show.

## The moral contingentist counterargument

As anticipated, Moral Contingentism allows for the metaphysical possibility of two duplicates that share all descriptive properties and yet differ in moral status, due to the metaphysical contingency of pure moral laws. This result is welcome, as it allows perdurantists to hold that if Dee had died 30 min earlier, then Dee-minus would have been a person, while maintaining that such a scenario is normatively impossible and therefore normatively distant, i.e., a countermoral world. This blocks Dee-minus from acquiring moral status through duplication, since the pure moral laws can differ there and thus ground moral status differently.

Thus, perdurantists can claim that there are one or more pure moral laws which normatively necessitate that only maximal aggregates of R-interrelated stages—i.e., persons such as Dee and Dum—have moral status.^21^ The precise formulation of these laws need not be specified: what matters is that, *if they hold*, they do so across every normatively possible world; the problem is that there is no metaphysical guarantee that they in the actual world. However, the Duplication Argument as Johnston presents it in the Dum-Dee scenario is effective precisely because it assumes that, standardly, persons, but not personites, have moral status in the actual world according to perdurantists (cf. Johnston, 2017, pp. 618–621). Thus, it is reasonable for those who adopt Moral Contingentism to assume that the pure moral laws necessitating precisely this distribution of moral status do indeed obtain in the actual world. With all the pieces now in place, the Moral Contingentist Counterargument to Johnston’s Duplication Argument runs as follows:

1. (All persons have moral status as a matter of normative necessity): For all normatively possible worlds *w* and possible objects *x*, if *x* is a person in *w*, then *x* has moral status in w.
2. (Within any non-empty subset of the metaphysically possible worlds where the same pure moral laws hold, all duplicates share moral status): For all metaphysically possible worlds *w* and *v* that share the same pure moral laws, and for all possible objects *x* and *y*, if *x* in *w* is a duplicate of *y* in *v*, then *x* has moral status in *w* iff *y* has moral status in *v*.
3. (Being a duplicate of a person in some normatively possible world is the only route for personites to have moral status): For all normatively possible worlds *w* and personites *x*, if *x* has moral status in *w*, then there exists a normatively possible world *v* and object *y* such that *y* is a person in *v*, *v* shares the same pure moral laws with *w*, and *x* is a duplicate of *y*.
4. (All personites have at least one possible person as a duplicate as a matter of metaphysical necessity, but all such worlds are countermoral): For all personites *x*, there exists a metaphysically possible world *w* and object *y* such that *y* is a person in *w* and *x* is a duplicate of *y*; however, all such worlds differ in their pure moral laws from the actual world and are therefore normatively impossible.

Therefore: All personites lack moral status as a matter of normative necessity.

To sum up, the Moral Contingentist Counterargument can effectively respond to Johnston’s Duplication Argument by retaining the metaphysical possibility that personites can be duplicates of persons, while holding that the worlds in which this occurs are countermoral and therefore normatively inaccessible. This ensures that personites lack moral status in all normatively possible worlds, including the actual world, since pure moral laws can differ across countermoral worlds and thereby ground moral status differently.^22^

## Objections and replies

Now, let me examine some of the possible objections to my Moral Contingentist strategy for perdurantists and the counterargument to Johnston’s Duplication Argument.

First, one of the most common objections to Moral Contingentism, already anticipated earlier, is the Authority Objection: morality must be metaphysically necessary, otherwise, it would fail to fulfill its action-guiding role. The normative authority of moral laws is typically thought to rest on their metaphysical necessity (cf. Väyrynen, 2021, pp. 2–4). Moreover, Moral Contingentism appears to allow for countermoral worlds such as worlds where enslaving other persons is permissible, which on Moral Necessitist views which take pure moral laws to be metaphysically necessary are ruled out as metaphysically impossible.^23^

To dispel the first worry, it can be replied that, while metaphysically contingent, in Moral Contingentism pure moral laws hold with normative necessity. Though weaker than metaphysical necessity, normative necessity is still expected to be sufficiently modally robust, such that pure moral laws hold not only at a single world but across several nearby worlds. In this sense, morality would still retain at least part of its normative authority. Moreover, in response to the second, related concern, countermoral worlds, such as one in which enslaving other persons is permissible, would be very distant in normative space and thus would not pose a threat to the normative authority within a given set of worlds where the pure moral laws prescribe the opposite.^24^

But the objector at this point could raise one of the stronger objections to Moral Contingentism: since the view is incompatible with Full Metaphysical Dependence (FMD), it allows for two metaphysically possible worlds to be descriptively identical yet differ normatively. Consider once again the Dum-Dee scenario, in which a person and a personite are descriptive duplicates yet differ in their normative properties. A world where Dee-minus, a personite, has moral status and Dum, a person, does not, is metaphysically possible, as it is descriptively identical to the actual world, but normatively impossible. While normatively distant, such a world could be metaphysically, and perhaps even nomologically, close. The objection to the perdurantist, then, is this: it would seem to be a matter of luck to be located in a world where persons, and so Dum, but not Dee-minus, have moral status, rather than in one where the opposite obtains.

Prima facie, the Luck Objection^25^ is strong. It is not difficult to imagine a metaphysically possible world in which persons have no moral status: consider, for instance, a countermoral world in which moral status is accorded exclusively to beings capable of telepathic communication, and in which such beings consider other sentient creatures, including human persons, as morally insignificant. Such a world would presumably be very distant from the actual world not only normatively but also nomologically: cognitive evolution would likely have followed a radically different path, and there would be widespread differences in descriptive facts. As a result, it would differ from our world in its laws of nature and possibly in its metaphysical structure as well, making it comparatively straightforward to classify as distant without any appeal to luck. By contrast, the case of a world in which Dee-minus has moral status while Dum does not, the scenario on which the objection turns, seems significantly harder to dismiss, as it would be a countermoral world that is not only metaphysically possible but also presumably nomologically very similar to ours, yet one in which personites have moral status while persons do not.^26^ This lends force to the Luck Objection, since Moral Contingentism, by its own lights, cannot appeal to descriptive differences to explain the variation in certain normative properties such as moral status, which are grounded in pure moral laws that are, by definition, independent of descriptive facts. It thus appears that the Moral Contingentist must bite the bullet of moral luck, or else risk their position collapsing into a reductio ad absurdum.

In response to similar concerns advanced by Dreier (2019, pp. 1406–1407), Rosen (2020, pp. 224–227) discusses the following case: suppose that in the actual world, @, Act-Consequentialism does not hold, so all possible worlds in which Act-Consequentialism holds are normatively impossible with respect to @. Suppose that we are cosmetic surgeons who must decide whether to operate on Sam to make him more beautiful, and that in @, the pure moral laws in place prescribe that it is permissible to operate on someone only if they agree to the surgery. Suppose that, indeed, the surgery would be the best course of action for improving Sam’s appearance and would thereby maximize utility in that respect, and that Sam agrees to have the surgery. Irrespective of the fact that the surgery maximizes utility, one should conclude that performing the surgery in @ is morally permissible, since the fact that Sam gives his consent is what grounds the permissibility of the surgery according to the pure moral laws in place in @. Now, consider these two distinct possible worlds:

- The same pure moral laws as in @ hold. All the descriptive facts are the same as in @, except that Sam does not agree to have the surgery.
- Act-Consequentialism holds. All the descriptive facts are the same as in @, except that Sam does not agree to have the surgery.

Intuitively, the friends of the Luck Objection would say that (*b*) appears to be at least as close as (*a*), if not more, since the surgery is permissible in (*b*) as it is in @, while in (*a*) it is not. But for Rosen the moral contingentist is safe from the objection for this reason: the fact that the act is permissible in (*b*) as it is in @, while in (*a*) it is not, does not make (*b*) closer than (*a*). In @, by contrast with (*b*), the fact that the surgery maximizes utility plays no grounding role, as it is not part of the grounding chain that ties the act’s permissibility to the pure moral laws in place. Similarly, in (*b*), the same could be said of the fact that Sam agrees to the surgery. Instead, the impermissibility of the surgery in (*a*) actually makes it closer to @ than (*b*), since the same normatively necessary factors—i.e., the pure moral laws—explain the opposite contingent normative facts regarding permissibility in both worlds. Therefore, (*b*) is a countermoral world with respect to both (*a*) and, more importantly, @, and thus poses no threat.

In the same vein, the perdurantist who adopts Moral Contingentism could defend their position by proposing the following case. In the actual world, @, the pure moral laws prescribe that persons have moral status, while personites do not. Suppose, as in Johnston’s example discussed in Sect. 2, that a person, Rita, must decide whether to undertake prudential self-sacrifice by enrolling in an intensive Hungarian language course. The act would maximize utility. However, the course requires one additional participant to commence, and no one else is willing to join. Rita asks her friend Sara to enroll with her, but she declines. Rita then considers secretly enrolling Sara, covering her share of the cost, in order for the course to proceed. Yet in @, an act is permissible only if it has the consent of all entities with moral status who are capable of consenting, or who have been, will be, or have the potential to be in a position to consent. Therefore, in @, prudentially self-sacrificing to learn Hungarian by enrolling in the course with Sara without her consent is impermissible.

This time, however, consider three distinct possible worlds:

(c) The same pure moral laws as in @ hold. Almost all the descriptive facts are the same as in @, except that Sara agrees to enroll. Learning Hungarian is permissible. The fact that Sara has moral status and consents plays a grounding role in explaining the permissibility of the act.
(d) Different pure moral laws from those holding in @ hold: personites, not persons, have moral status. All the descriptive facts are the same as in @. Learning Hungarian is permissible. The fact that Sara consents and that personites have moral status but cannot consent plays no grounding role in explaining the permissibility of the act.
(e) Different pure moral laws from those holding in @ hold: personites, in addition to persons, have moral status. All the descriptive facts are the same as in @. Learning Hungarian is impermissible. The fact that Sara has moral status and does not consent plays a grounding role in explaining the impermissibility of the act, while the fact that personites have moral status but cannot consent plays no grounding role.

If one accepts Rosen’s line of argumentation, one must conclude that (*c*) is closer to @ than either (*d*) or (*e*). Even if a proponent of the Luck Objection were to argue that one should disregard differences in pure moral laws when assessing the closeness of worlds—e.g., on the grounds that countermoral worlds can be similar in other respects (nomological, metaphysical, or even epistemic), and thus there is a kind of luck involved in being located in @, albeit not strictly moral—one should note that the inability of personites to consent, a key point in Johnston’s formulation of the Personite Problem (cf. Johnston, 2016, pp. 631–634, 2017, pp. 207–209), seemingly never plays a role in grounding permissibility in any of the worlds examined, even in those where they have moral status. For this reason, this fact does not affect the closeness of the worlds examined to @: it could do so only if personites had, at any point, the capacity or potential to consent, but if that were the case, they would qualify as persons, and thus the relevant fact would no longer be the same.Thus, the perdurantist who endorses Moral Contingentism can conclude that, mutatis mutandis, worlds like (*c*)—in which persons have moral status and personites do not—will always be closer to @ than worlds like (*d*) and (*e*), in which personites have moral status, and can therefore mount a defence from the Luck Objection.

Moreover, other metaethical views are not immune to the Luck Objection, albeit to different degrees: for example, consider Moral Necessitism, where pure moral laws are metaphysically necessary and thus fact-independent in the broadest possible sense, contrary to Moral Contingentism, where fact-independence is set-relative. Arguably, many versions of Moral Necessitism still allow for many metaphysically possible worlds that are nonetheless incompatible with the pure moral laws: suppose that the pure moral laws prescribe libertarian free will, but in many worlds it is nomologically impossible. Then, it would also be a matter of luck to be located in a world where libertarian free will is nomologically possible. Some of these worlds, in fact, could be duplicates in every other respect, and thus relatively close, apart from some differences in the laws of nature that make libertarian free will nomologically impossible, even though it remains morally prescribed. But we could also do without considering nomologically impossible worlds, and instead focus on possible ones: the Luck Objection would perhaps be weaker, but still effective to a degree. Consider a world where the gravitational constant is slightly stronger than in the actual world. Such a small change would not make this world radically distant, especially if we assume that in other respects this world is almost a duplicate of the actual world. However, everyday physical activities would likely require somewhat more effort. In such a world, complying with certain moral duties, such as standing up to offer one’s seat to an elderly person on a crowded bus, would involve a greater physical burden compared to our own, while the pure moral law prescribing that one ought to give up their seat would remain unchanged. This suggests that even under Moral Necessitism, the ease or difficulty of fulfilling moral obligations could depend on luck—this time, *nomological* luck. The only kinds of metaethical stances that seem capable of evading the Luck Objection are those which are fully naturalist and allow moral laws to vary in accordance with descriptive facts alone. Arguably, the world with a slightly stronger gravitational constant just described would involve an adjustment of the moral laws in line with the laws of nature in place. However, these same stances fall prey to the already mentioned Authority Objection: under such views, moral laws would be neither metaphysically necessary nor even normatively necessary, but merely nomologically necessary, which is plausibly an even weaker form of necessity than normative necessity.

Thus, it seems that most metaethical views are, to different degrees, prone either to the Luck Objection or to the Authority Objection, and in some cases to both. Anyhow, Moral Contingentism does not seem to fare worse than others, for the reasons I have tried to provide, since it is possible to address the Authority Objection and dispel some of the worries connected to the Luck Objection.

## Concluding remarks

In this paper, I have argued that perdurantists who adopt Moral Contingentism can directly counter Johnston’s Duplication Argument for the Personite Problem. I have contended they can hold that personites could have been persons in some metaphysically possible worlds, while denying that such worlds pose a threat, since they are countermoral and thus normatively impossible. Although denying that personites are duplicates of persons may seem the more straightforward strategy, my aim has been to offer an alternative for perdurantists who wish to resist Johnston’s argument without abandoning the counterfactual duplication-based point altogether—whether because they are reluctant to make contentious claims about which descriptive properties personites lack, or because they do not want to revise the duplication principle itself. In any case, for perdurantists committed to preserving that point, the cost of endorsing a non-standard view of morality may well be a price worth paying.

## Data Availability

Not applicable.

## Footnotes

1 The R-relation is often taken to be psychological continuity, following Parfit (1984), but other kinds of relations, such as physical continuity, are admissible. Here, I stay neutral on its nature, holding that it is whichever appropriate relation should hold between the temporal parts of a person.

2 See Lewis (1976) for a classical statement of the view. More recent ones can be found in Russo (2022) and Tullio and Soriani (2024).

3 See Builes and C. Hare (2023). Following Longenecker (2020), here the locution “for any arbitrary collection” employs a tenseless quantifier.

4 See Kovacs (2024) for a differing view on this.

5 Johnston (2016, pp. 212–214, 2017, pp. 623–624). See also Zimmerman (2003), Olson (2010) and Taylor (2013) who each independently raised concerns similar to Johnston’s.

6 Following Johnston (2016, 2017), I reserve the pronoun “it” for personites.

7 While Lewis sometimes appears to allow duplication both within and across worlds solely on the basis of shared intrinsic properties (cf. Langton & Lewis, 1998; Lewis, 1986, pp. 61–63), he elsewhere claims that world-mates must also share all extrinsic properties to count as duplicates (cf. Lewis 1983a, p. 199, 1983b, p. 356, n. 16; cf. also Pigden & Entwisle, 2012, p. 6). This does not render them indiscernible, since duplication is defined by intrinsic *qualitative* properties, and world-mates can still differ in *non-qualitative* respects. The rationale for this intra-world restriction becomes clear when considering mereological relations: if duplication within a world required only intrinsic similarity, then Dee-minus could count as a duplicate of Dum in the actual world @, as could uncountably many overlapping personites of Dee differing from Dum only in extrinsic respects; duplication would thus be trivialized. Similar issues do not arise with inter-world duplication which allows us to hold intrinsic properties fixed while varying extrinsic ones, including world-relative mereological properties. Thus, Dee-minus, though non-maximal in @, may have a maximal counterpart Dee* in another world *w* who shares all her intrinsic properties and can be a duplicate of Dum. Likewise, Dee-minus*, a personite of Dee* who ceases to exist two hours earlier, can also qualify as a duplicate of Dum, since they inhabit different worlds and are unaffected by each other’s mereological context. Hence, personites cannot have world-mate persons as duplicates, but may have persons as duplicates at other worlds.

8 See Lewis (2001, pp. 16–17) and Starr and Kocurek (2019, Sect. 5) for the counterfactual rule. In the last section of the paper, Sect. 6, I present several reasons to support the view that the world in which Dee-minus has a possible person as a duplicate is a countermoral world, and thus very distant in normative space, based on multiple factors, including Rosen’s (2020) interpretation of Lewis’s (1979) Similarity Model for determining the closeness of worlds, which is itself influenced by Kment (2006).

9 This formulation owes to Kaiserman (2019, p. 217) and Seymour (forthcoming, p. 4). See also Kowalczyk (2022, p. 4) for another version of this argument.

10 Kaiserman’s (2019) counterargument belongs to a different category, since it does not aim at preserving Perdurantism but argues for the adoption of the *Stage-theory* instead—the view that persons are identical to instantaneous person-stages and persist thanks to a counterpart relation—and thus concludes that personites cannot be persons, since they are neither instantaneous nor can they be duplicates of instantaneous entities.

11 See Tannenbaum and Jaworska (2018, 2019) for a discussion of the grounds of moral status and personhood.

12 Cf. Väyrynen (2021, pp. 2–4). I discuss this objection, the Authority Objection, and possible ways to dispel it in Sect. 6.

13 Thanks to two anonymous referees for prompting this specification.

14 See Väyrynen (2021, pp. 1–2). As Rosen (2021, p. 258) argues, any example is prone to objections once specified, likely because it is difficult to formulate them without including some “impure” element, so to speak.

15 Lange (2018, pp. 170–171) tries to capture this idea expressed by Fine by quoting the following paragraph, suggesting that the same would apply, for Fine, to normative necessities with respect to metaphysical ones, as is the case with nomological necessities with respect to the latter: “There appears to be an intuitive difference to the kind of necessity attaching to metaphysical and natural necessities (granted that some natural necessities are not metaphysical). The former is somehow ‘harder’ or ‘stricter’ than the latter. If we were to suppose that a God were capable of breaking necessary connections, then it would take more of a God to break a connection that was metaphysically necessary than one that was naturally necessary” (Fine, 2002, pp. 278–279).

16 There is also the possibility that a world in which torturing for fun is permissible is ultimately metaphysically impossible in virtue of facts about the *essence* of permissibility. See Morin-Martel (2024, p. 3), as well as Rosen (2021, p. 263), for further discussion. But this does not undermine the point that there is no prima facie metaphysical contradiction in describing such a world prior to any appeal to essences, and invoking essences would require an additional commitment that not all Moral Contingentists may be willing to accept; so it is, prima facie, metaphysically possible. More generally, it is harder, not easier, to show that torture-permissive and similarly abhorrent worlds are metaphysically impossible. Our strong intuition that such worlds are impossible is not, by itself, reliable evidence of genuine metaphysical impossibility: as Hills (2009, pp. 169–174) argues, our resistance to imagining them is best explained by a desire-based form of imaginative resistance. Hattiangadi (2018, p. 595) similarly suggests that the differing imaginability of abhorrent worlds and apparently innocuous cases, such as a world where benevolent lies are always wrong, is best explained by the varying strength of our moral motivation, not by any difference in metaphysical impossibility. It is therefore more parsimonious, within a Contingentist framework, to explain the apparent impossibility of abhorrent worlds through normative distance rather than by incurring additional metaphysical commitments—e.g., essences—that other positions—e.g., Necessitism—may require. Thanks to an anonymous referee for prompting this clarification.

17 By contrast, *impure* moral laws are those that are at least partly grounded in descriptive facts. For example, the impure moral law “it is wrong to kick dogs” is not purely normative but also depends on descriptive facts about dogs—viz., their capacity to feel pain. If dogs were not able to feel pain, then, at least under most views, kicking them would not carry the same moral significance. Impure moral laws are thus grounded in a combination of pure moral laws and non-normative facts, while pure moral laws may themselves be grounded only in more fundamental pure laws, thereby forming a hierarchical structure (see Rosen (2017, p. 141). However, see also Bader (2017, pp. 117–118) for a different proposal: pure moral laws would not directly do any grounding work but would instead govern the grounding relations connecting descriptive properties to normative properties.

18 In Sect. 6, I further argue for the normative distance of countermoral worlds, also relying on Rosen (2020, pp. 224–227).

19 Not all normative elements need to be grounded in pure moral laws: apart from pure moral laws themselves, which may be ungrounded, some such as pro tanto reasons, instrumental norms, and thick evaluative ones like courageousness or cruelness, may depend on contingent social practices, agent-relative goals, or context-specific standards. The claim here concerns only those normative properties like moral status which are *substantive* and foundational to moral evaluation, in that they determine what counts as permissible, impermissible, obligatory, or worthy of respect (cf. Morin-Martel, 2024, p. 3); Rosen (2021, p. 269, n. 15).

20 In general, the more the properties in *C*, the closer one moves toward a naturalist position (see Stamatiadis-Bréhier (2025a, p. 20) for a similar proposal formulated in terms of supervenience). This is to remark that, while Moral Contingentism as I have presented it will be particularly appealing to perdurantists who are not afraid to embrace full-fledged non-naturalism, it may still appeal to those who do not wish to stray too far from naturalism.

21 This is compatible with the idea that the pure moral laws prescribe that maximal continuants in general have moral status while non-maximal ones do not, thereby extending the moral domain to include, for instance, animals—maximal aggregates of animal-stages connected by whatever relation one takes to be appropriate for linking the temporal parts of the same animal—and so on.

22 It is worth underlining that my Counterargument is formulated within a Law-based version of Moral Contingentism, specifically Rosen’s (2017, 2020, 2021), on which pure moral laws are fact-independent (in the sense described in Sect. 4) and partially ground the ethical facts. It does not straightforwardly extend to Particularist versions, on which there are no pure moral laws governing base-ethical facts, since any general pattern among those facts is explained by the particular ethical facts themselves, nor to Neo-Humean versions, on which moral lawhood is grounded in, and in a sense summarises, the distribution of base-level properties at a world. Accordingly, both of these versions may not be able to guarantee the possibility of descriptively similar or identical worlds that differ in pure moral laws, and so cannot guarantee the normative distance on which the present Counterargument depends. See, respectivelyHills (2009); Stamatiadis-Bréhier (2025b), Cannon (2025). Thanks to an anonymous referee for prompting me to specify this.

23 See Dreier (2019, pp. 1394–1396). Blackburn (1988) and R. Hare (1952) are among the classical references.

24 The strategy mentioned above (n. 16), that such a world might be ruled out by the essence of permissibility, can work here if one buys into that essentialist view. But, as I also anticipate in the same footnote, normative distance alone suffices, since it is still robust enough to ensure that we would be safe from such worlds: in a world *w* in which having slaves is morally permissible, the pure moral laws grounding that fact would be radically different from those of the actual world @, so it is reasonable to assume that the normative differences would not concern only that normative fact but others as well, making *w* and @ even more distant. So, even in the more remote hypothesis that @ and *w* were descriptively identical, the differences in normative facts alone would make sure that *w* does not constitute a threat, as I further argue below, drawing also on Rosen (2020, pp. 224–227). I thank an anonymous referee for drawing my attention to this point.

25 My formulation and discussion of the objection owe to Dreier (2019) who first advanced a series of related concerns toward Moral Contingentism, and Rosen (2020) who tentatively addressed some of them, as well as to Morin-Martel’s (2024) reconstruction of this debate.

26 Appealing to essences to rule out such countermoral worlds (see nn. 16, 24) is unavailable if one wishes to preserve their metaphysical possibility. If one argues that persons and personites are so essentially—see Tullio and Soriani (2024, p. 17) for a similar move—such worlds would be metaphysically impossible. A more restricted version could preserve their metaphysical possibility by endorsing a distinction between *metaphysical* and *normative* essences (a distinction which Morin-Martel (2024, p. 3), drawing on Rosen (2021, p. 263), hints at): one might be normatively essentially a person, allowing thus the metaphysical possibility of being a personite outside the set of normatively possible worlds (and vice versa). Both strategies, however, require further commitments to essences which, as remarked, not every perdurantist would be willing to take.
